# Acculturation and English proficiency among adult newcomer immigrant ESL learners in Canada across language instruction methods

**DOI:** 10.1177/13670069251357191

**Published:** 2025-07-26

**Authors:** Ali Jasemi, Alexandra Gottardo, John W. Schwieter

**Affiliations:** Wilfrid Laurier University, Canada; Wilfrid Laurier University, Canada; McMaster University, Canada

**Keywords:** Acculturation, ESL, immigrant communities, language pedagogy, second language acquisition

## Abstract

**Aims and objectives:**

This study investigated the impact of different instructional environments on learners’ acculturation and English language skills among adult English as a Second Language (ESL) learners in Canada to address this gap in the literature.

**Design:**

Using a series of qualitative and standardized measures, a robust mixed-methods approach was taken to explore the relationships among those variables. This study included 56 participants attending community-based English classes with learners mainly socializing with other ESL learners and university-based English for Academic Purposes (EAP) courses, where ESL learners were exposed to other students fluent in English.

**Data and analysis:**

Quantitative and qualitative analyses were conducted to assess English reading comprehension, English vocabulary, level of mainstream acculturation, heritage enculturation, and social adjustment.

**Findings:**

The findings revealed that EAP courses were associated with significantly higher English proficiency and acculturation rates among ESL learners compared to community-based programs. Furthermore, acculturation emerged as a significant predictor of English reading comprehension across both educational settings. Learner satisfaction was noted for each instructional program.

**Originality:**

Previous studies have highlighted the significance of acculturation and social integration in facilitating language acquisition among immigrants and international students. However, the Canadian context regarding adult ESL learners remains underexplored.

**Significance/implications:**

These findings offer insights for policy and practice in multicultural educational settings and highlight the need for ESL programs to further integrate social and cultural immersion elements within academic structures to facilitate successful language acquisition and acculturation.

## Introduction

As globalization fosters diverse, multilingual societies, understanding the dynamics of second language acquisition becomes increasingly critical, especially in immigrant-receiving countries such as Canada. Although numerous studies in the literature examine second language acquisition among children, fewer studies are conducted with adult learners (Baynham & Simpson, 2010). Furthermore, even fewer studies are available regarding adult English as a Second Language (ESL) learners in the Canadian context, as an immigration country where many of those immigrants come from non-English-speaking countries (Statistics Canada, 2022). This study aims to fill the existing research gap by examining how different English instructional methods affect adult ESL learners’ language proficiency and acculturation in the Canadian context. Specifically, we investigate the comparative effectiveness of academic versus community-based English instruction, the relationship between acculturation and language proficiency, and English learners’ experiences and outcomes within these educational frameworks.

## Background

The ability to speak more than one language has sparked an interest in examining the processes involved in second language (L2) acquisition. Sequential L2 acquisition (i.e., the process of learning an L2 after the first language (L1) has been largely acquired) is common in countries with significant immigration, such as Canada and the United States. For instance, in Canada and the United States, approximately 20% of the population speaks more than one language at home (Statistics Canada, 2022; Dietrich et al., 2022). This rate is even higher in European countries, where approximately 60% of citizens report being able to communicate in at least one foreign language (Mickan et al., 2019). Therefore, it is not surprising that studies are conducted to examine L2 development in children, adolescents, and adults in a secondary or postsecondary school setting (Mitchell et al., 2013). However, there is a lack of research investigating the effects of pedagogical methods and contexts on L2 development among newcomers to Canada.

Several factors may play a role in adults’ L2 learning, which may differ from learning in adolescents. For instance, previous studies have demonstrated the importance of contextualized learning for adult language learners. Purcell-Gates et al. (2002) suggested that using real-life, relevant materials in the target language is related to a higher involvement in reading and writing in that language and greater engagement in more complex texts. Moreover, learner autonomy may play an essential role in L2 acquisition for adult English learners. As Holec (1981) describes, such autonomy refers to an individual’s role in taking charge of their learning, which may be elicited by goal setting, choosing the content to be studied, redefining the goals throughout formulation, and, in some instances, the programs of the study.

Another important factor related to language acquisition, especially for immigrants, is acculturation. According to Berry (1980), acculturation is the process by which a person adapts to cultural norms in new contexts. During the acculturation process, newcomers integrate their original culture with the norms of a new one. The acculturation process has been shown to affect L2 learning significantly (Schumann, 1986) with more successful acculturation playing a role in better L2 learning outcomes, including enhanced language proficiency (Jiang et al., 2009), as new immigrants settle in a new country and potentially adopt new cultural norms.

Contextualized learning may also affect adult learners’ success and improvement in their L2 skills. Contextualized learning is rooted in constructivism (Brown et al., 1989; Vygotsky, 1978). According to Berns and Erickson (2001), an individual can adapt and apply the learned subjects to new situations which are different than the original context. In this light, English learners’ involvement with a new culture, known as acculturation, may enable them to enhance their language skills by participating in cultural activities related to the language they are learning. This process may relate to the nature of the content being learned by and provided to adult language learners. For instance, a language learner attending an English for Academic Purposes (EAP) course may be exposed to more academic content and the target language’s mechanics rather than day-to-day phrases or specific vocabulary that may be emphasized in government-funded L2 classes.

Many studies emphasize cultural aspects of language learning and the importance of socialization (Steen et al., 2017), particularly when immersed in an L2 outside of one’s home country (Jackson et al., 2020; Jackson & Schwieter, 2019; Schwieter et al., 2021, Forthcoming; Schwieter & Ferreira, 2020; Schwieter & Kunert, 2012). In addition to formal pedagogical approaches, teachers’ attitudes and beliefs may also impact L2 learners’ learning outcomes (Mustafa et al., 2019). Evidence also suggests that literacy-oriented programs are not suitable for all migrants in promoting acculturation (Nieuwboer & van’t Rood, 2016). For example, some studies suggest that structured learning environments may be more beneficial for refugees in improving their L2 skills, while other immigrants might find informal exposure more helpful (Kosyakova et al., 2022). Furthermore, many new language learners find the cultural components of language learning, such as acculturation, essential to language acquisition (e.g., Brunsting et al., 2018; Young & Schartner, 2014).

Other evidence suggests that the interplay between the language acquisition process and socialization seems to be highly interrelated, as the migrants’ competency in the host country’s language depends on their ability to use the cultural tools and their capacity to apply language fluency within various cultural contexts (Soto Huerta & Pérez, 2015). These abilities form part of speakers’ communicative competence (Hymes, 1966). Therefore, learning methods emphasizing academic aspects of language learning may exclude cultural components as significant contributors to language acquisition. Likewise, the relationships between the language learning methods and cultural aspects of language acquisition may differ based on the host country’s context.

In the Canadian context, immigrants who are English learners have access to various programs and resources such as Language Instruction for Newcomers to Canada (LINC), English as a Second Language (ESL), International English Language Testing System (IELTS) classes, and Test of English as a Foreign Language (TOEFL) preparation classes funded by the government. The ESL and LINC classes offered in communities across Canada focus on both language and cultural components of resettlement. Many ESL and LINC classes use real-world content such as job postings, news content, and other similar documents to contextualize study material. These programs are designed to teach basic and conversational language skills to facilitate employment and integration into society. English learners in Canada may also attend ESL courses in universities and community colleges. These classes are mainly geared toward English for Academic Purposes (EAP) and aim to prepare the students to complete language tests for admission to higher education (e.g., IELTS and TOEFL). Many postsecondary institutions require minimum scores on the IELTS or TOEFL for admission to their programs. Finally, some newcomer immigrants may take a different path and not attend language classes in Canada by choice or necessity. Instead, they may start learning the L2 through social interactions at work or in the community after settling in Canada. This process of informal language learning is also known as interactional feedback, which occurs during the acculturation process (Nassaji, 2015). During this process, an individual with low levels of L2 proficiency may learn the language by receiving feedback throughout their interactions with individuals with higher skills in the target language. This form of learning is often called “negotiation and modification” and helps address common linguistic and communication problems (Nassaji, 2015).

Previous research has shown that cross-language transfer may play a role in language learning outcomes (Riches & Genesee, 2006). For instance, Zhang et al. (2024) conducted a longitudinal study on cross-linguistic transfer of phonological and morphological awareness among Spanish-English and Chinese-English heritage-speaking children. The results demonstrated differential patterns of cross-linguistic transfer, such that the Spanish-English bilinguals had stronger associations between morphological awareness skills between the two languages, whereas Chinese-English bilinguals showed stronger associations between morphological and word reading skills. Similarly, in another study, Isphording (2014) found that cross-language transfer from immigrant adult learners’ L1 to the host country’s language was influenced by both linguistic similarity and parental education. Together, these findings reflect the distinct influences that heritage languages have on L2 acquisition (see also Durgunoǧlu, 2002). In addition, some studies have found differences in language learning outcomes when comparing alphabetic languages, such as Farsi, and non-alphabetic languages, such as Chinese (Akamatsu, 1999). Akamatsu’s findings emphasize the important role of different L1s on word recognition and reading efficiency in L2.

Considering the role of L1 on L2 acquisition and proficiency, the potential effects related to the unique characteristics of L2 learning environments in Canada should be considered. One of the significant characteristics of the ESL learning environment for immigrants in Canada is the diversity of languages among individuals in learning situations. In some cases, the foreign languages English learners speak may be homogeneous. For instance, according to the California Department of Education (2025), over 80% of the English learners enrolled at California schools spoke Spanish at home. Therefore, the method of English language instruction may be geared toward accommodating Spanish-English learners. However, in Ontario, Canada, linguistic diversity is significantly broader (Statistics Canada, 2022). According to census reports, some of the most frequent languages spoken by immigrants in the country include Mandarin, Cantonese, Punjabi, Arabic, Spanish, and Farsi (Statistics Canada, 2017). These newcomers can access government-funded English language programs such as LINC classes, irrespective of the learners’ L1 background (Government of Canada, 2018).

## Present Study

Despite extensive research on child and adolescent language learning, research on English learning in adulthood remains less explored. L2 learning in adult newcomer immigrants is as a crucial factor for successful social integration, employment, and academic achievement among immigrants remains less explored. This study aims to compare the impact of EAP and community-based English instruction on adult learners in Canada, with a focus on acculturation and language proficiency.

The rationale for comparing these groups of learners is rooted in the hypothesis that varying pedagogical environments may reveal different effects on language proficiency and social adjustment outcomes among newcomer migrants. Examining these different educational settings, the study aims to determine the difference in learning paths in relation to adult ESL learning in Canada.

This study employed statistical controls to account for variations in educational and cultural experiences and their impact on language learning and acculturation. Therefore, the present study examines the effects of different pedagogical methods and contexts using qualitative and quantitative methods. Newcomers’ L2 learning outcomes and experiences may offer a better understanding of the L2 acquisition processes specific to Canada. The following research questions are investigated:

*RQ1. Does the group learning EAP have different English skills than the Community Group (CG)?*
*RQ2. Does the EAP group differ in acculturation (i.e., the process by which a person adapts to cultural norms in new contexts) or enculturation (i.e., the gradual acquisition of characteristics and norms of another culture or group) from the CG group?*
*RQ3. Are there relationships among different socio-cultural and linguistic variables for each group?*
*RQ4. Does acculturation or vocabulary knowledge predict reading comprehension for EAP and CG learners?*
*RQ5. What are English learners’ reported experiences with different teaching methods and social interactions in Canada?*

## Method

### Design

To strengthen the analysis of instructional effects, acculturation, and other relevant variables, this study employs a mixed-methods approach (Riazi & Farsani, 2024), pairing self-reported data with standardized quantitative measures for English language proficiency and acculturation. While retro-spective self-report and qualitative data have limitations, such as biases in reporting, they offer valuable insights into the learners’ perceptions and experiences to supplement the data gathered using quantitative measures. This information further helps us understand the subjective aspects of language learning and acculturation.

### Participants

The Research Ethics Board approved this study at the research institution. A total of 56 participants were recruited from Southern Ontario. All participants provided their informed consent prior to participating in the study. The participants were between 17 and 45 years old and had immigrated to Canada 2 to 5 years prior to the present study (*M* = 3.35, *SD* = 1.09) (see Table 1). Participants were recruited from two distinct educational settings to ensure a diverse representation of adult ESL learners, which included university-based EAP courses (EAP) (*N* = 29, 20 females and nine males) and community-provided English classes (CG) such as LINC or ESL classes (*N* = 27, 19 females and eight males). This approach comprehensively compared formal academic learning environments and more informal, community-driven instruction methods. The CG participants were recruited using flyer ads.

**Table 1.** Participants’ duration of stay and age of arrival in Canada.

	Minimum	Maximum	*M*	*SD*
Duration of residence in Canada	2	5	3.35	1.09
Age of arrival	17	45	28.85	8.41

In addition, a snowball sampling method was used by asking the participants who had already completed the study to invite their peers who met the requirements to participate. CG participants received a monetary compensation of $15 for participating in the study. EAP participants were recruited through the Psychology Research Experience Program (PREP), designed to provide students with firsthand experience with participation in scientific research. These individuals received academic credit for participating, which could be used as a bonus mark in relevant psychology courses. The participants included in the CG and EAP groups were from various regions across the world. Individuals in the CG group had varying levels of education, while the most common level of education among the EAP group was some college or university. The study sample is characterized by a wide ethnic range (see Table 2) among speakers of various L1s (see Table 3). The diversity in occupations and educational backgrounds, from high school to professional degrees, provides a comprehensive perspective on ESL learning across different socio-economic status backgrounds.

**Table 2.** Participants’ socio-demographic characteristics.

	English for Academic Purposes Group		Community Group	
	*N*	%	*N*	%
Ethnicity				
Eastern European	4	13.79	2	7.41
Western European	5	17.24	2	7.41
Middle Eastern	3	10.34	15	55.56
South Asian	3	10.34	8	29.63
African	1	3.45	0	0
East Asian	9	31.03	0	0
South American	4	13.79	0	0
Highest education level				
Some high school	0	0	2	7.41
High school diploma	11	37.93	5	18.52
Some college or university	14	48.28	3	11.11
College degree	3	10.34	6	22.22
Undergraduate degree	1	3.45	3	11.11
Graduate or professional degree	0	0	8	29.63

**Table 3.** Participants’ first language.

L1	*N*
Albanian	3
Arabic	1
Bengali	1
Farsi	16
French	2
Gujarati	8
Hindi	1
Italian	5
Korean	1
Mandarin	4
Polish	1
Portuguese	2
Punjabi	1
Romanian	1
Spanish	2
Swahili	1
Tagalog	3
Tibetan	1
Turkish	1
Ukrainian	1

### Materials

The participants were asked to complete a series of quantitative and qualitative measures to assess their English reading comprehension, English vocabulary, level of mainstream acculturation, heritage enculturation, and social adjustment.

#### Acculturation

The acculturation measure aimed to explore participants’ language use and preferences regarding cultural affiliations and activities. This measure is adapted from the Acculturation Rating Scale by Cuéllar et al. (1995). The goal of this scale is to examine the participants’ level of involvement with either Anglo-Canadian culture or their heritage culture. Each participant read 26 statements about activities and was then asked to express the frequency of involvement in each activity on a five-point scale ranging from 1 (*not at all*) to 5 (*almost always*). Items included selecting preferences for socialization, leisure activities, foods, and so on. For instance, some items included in this measure were as follows: “I think of myself as Canadian” or “I socialize with peers from my heritage culture.” Each item included on this scale was comprised of one item addressing mainstream Canadian culture. In contrast, another item addressed the same construct regarding the participant’s affiliation with the participant’s heritage culture.

#### Reading comprehension

The Woodcock–Johnson (W-J) III Tests of Achievement Passage Comprehension subtest (Woodcock et al., 2001) was used to assess reading comprehension. The participants were tested regarding their ability to mentally represent the passage they read. The participants were asked to extract the text’s meaning and to process the concepts from the passage. The participants were then required to fill in the blanks with a single word to complete the sentence based on their comprehension of the passage they read. The initial passages include short, simple passages. A stopping rule was implemented such that participants were asked to stop after six consecutive incorrect responses or when they were unable to fill in six consecutive blanks.

#### Vocabulary knowledge

The Vocabulary Size Test (VST) by Nation and Beglar (2007) was used to test participants’ vocabulary knowledge. The participants are provided with a word in a brief, uninformative sentence. Each of the 100 sentences has four options, and participants are asked to select the option representing the meaning of that specific target word within the given context. For example, for one of the test items, the participants are asked about the meaning of the word “figure” within a context where “figure” would mean a number. In this case, the participants are provided with a test item as follows: “Figure: Is this the right <figure>?” The participants are then presented with four options to choose from. The choices would be: “a) answer, b) place, c) time, d) number.” To address the sample size issues, mainly related to the smaller number of participants who completed the vocabulary measure, the scores for both groups are combined for this research question. Analyses were performed to ensure the assumptions about homogeneity of variance were met which will be discussed further under the section “Method.”

#### Demographic and language experience questionnaire

The participants were asked to report demographic information such as age, ethnicity, highest level of education, and perceived fluency in English and their heritage language (using a 10-point scale). They were also asked about their length of time in Canada and their previous English experiences. They were asked about different language instruction methods and pedagogies and their social interactions, including the language used for those social interactions and the cultural contexts of those socializations.

#### Qualitative questionnaire

In the qualitative questionnaire, participants were asked to report their experience attending English classes using various instructional methods. For instance, the participants were asked what type of English classes they had attended in Canada. They were also asked to explain their experiences participating in these programs. The questionnaire also elicited information about participants’ affiliations with their heritage and mainstream Canadian cultures to expand on quantitative data. The questions were open-ended and, thus, allowed participants to elaborate on their opinions and experiences with the instructional contexts.

### Procedure

The participants attended the experimental session individually, and data were collected online using Qualtrics. Each participant received an individual anonymized link that could be used to complete the study. The consent form was also provided on the same platform prior to the data collection. The participants were asked to complete the measures after consenting to be part of the study. After the data collection, the participants were provided with a separate page where they could enter their contact information, if they opted to receive monetary compensation. All the questions included in the measures were programmed according to their original delivery methods, such as Likert-type scales, fill-in-the-blanks, or multiple-choice answers. The participants were asked to type in the answers to the questions requiring written responses.

At the end of the experimental measures, the participants were provided with a debriefing message, letting them know about the purpose of the study and the researchers’ contact information if they were interested in the study’s results. None of the measures had time limits, and the participants could take as much time as needed to complete them. Stopping rules were used for some of the measures (see Materials). The participants were instructed to avoid using dictionaries or translation services unless they required assistance to understand the instructions provided for the study. The responses were exported from Qualtrics and analyzed using SPSS software at the end of the data collection process.

## Results

The findings were analyzed using a mixed-methods design. Initially, group comparisons were conducted to analyze differences between EAP and CG learners. Then, further within-group analyses were conducted to examine the correlations. The results of the correlations informed the regression analyses. All central tendency statistics were calculated, and no ceiling or floor effects were detected. Statistical analyses, including analysis of variance (ANOVA), regression, and chi-square tests, were applied in response to the data’s distribution and measurement scales and the nature of the available data. Appropriate tests were conducted to ensure the homogeneity of variance for all the comparative analyses.

Furthermore, results are drawn from two approaches to complement the findings. Quantitative analyses were conducted regarding language proficiency and acculturation, followed by qualitative insights into participants’ experiences. Further details based on specific research questions are provided below.

### Does the group learning EAP have different English skills than the CG?

A one-way ANOVA analysis compared the differences between the EAP and CG groups’ English vocabulary knowledge and reading comprehension. For English vocabulary knowledge, EAP learners scored significantly higher (*M* = 62.87, *SD* = 15.77) than CG learners (*M* = 44, *SD* = 15.98), *F*(1, 25) = 9.23, *p* < .01. Regarding English reading comprehension, the EAP group also scored significantly higher (*M* = 20.18, *SD* = 4.45) than CG learners (*M* = 14.68, *SD* = 4.02), *F*(1, 50) = 21.72, *p* < .001 (see Table 4).

**Table 4.** Between-subject comparisons for English language variables.

	English for Academic Purposes Group		Community Group		
	*M*	*SD*	*M*	*SD*	*F*
English Passage Comprehension	20.18	4.45	114.68	8.85	21.72**
English Vocabulary Knowledge	62.87	15.77	444.00	15.98	9.23**

* *p* < .05. **p < .01.

### Does the EAP group differ in acculturation or enculturation from the CG group?

A one-way ANOVA analysis compared the acculturation and heritage enculturation rates between EAP and CG learners. For acculturation into Canadian culture, the EAP learners had significantly higher scores (*M* = 43.86, *SD* = 7.42) than CG learners (*M* = 35.70, *SD* = 7.74), *F*(1, 54) = 16.20, *p* < .001. Moreover, CG learners had a significantly higher heritage enculturation rate (*M* = 51.14, *SD* = 8.99) than EAP learners (*M* = 44.20, *SD* = 8.77), *F*(1, 54) = 8.53, *p* < .01 (see Table 5).

**Table 5.** Between-subject comparisons for acculturation and enculturation variables.

	English for Academic Purposes Group		Community Group		
	*M*	*SD*	*M*	*SD*	*F*
Acculturation	43.86	7.42	35.70	7.74	16.20 **
Enculturation	44.20	8.77	51.14	8.99	8.53 *

* *p* < .05. **p < .01.

### Are there relationships among different socio-cultural and linguistic variables for each group?

Bivariate correlational analyses were performed to explore the relationships among different socio-cultural and linguistic variables. The results for each group are discussed below.

#### Community English learners

Pearson’s correlation coefficients were computed to examine relationships among acculturation, heritage enculturation, English vocabulary knowledge, and English passage comprehension. There was a positive relationship between acculturation and passage comprehension, *r*(25) = .401, *p* < .05 (see Table 6).

**Table 6.** Correlational matrix for linguistic status and cultural measures.

	Vocabulary	Reading comprehension	Acculturation	Enculturation
Vocabulary	—	.537	.053	−.433
Reading comprehension	.767**	—	.401*	−.216
Acculturation	.366	.623**	—	−.276
Enculturation	.262	−.029	−.039	—

*Notes.* Results for the English for Academic Purposes Group are entered below the dashes, and the Community Group above the dashes.

* *p* < .05. **p < .01.

#### EAP learners

The Pearson correlation coefficients were computed to examine relationships among acculturation, heritage enculturation, English vocabulary knowledge, and English passage comprehension. There was a positive relationship between acculturation and passage comprehension *r*(27) = .623, *p* < .01. Moreover, the results showed that English passage comprehension was highly correlated with vocabulary knowledge *r*(27) = .767, *p* < .01 (see Table 6).

### Are acculturation or vocabulary knowledge uniquely related to reading comprehension for this sample?

For this research question, the effects of acculturation and English vocabulary knowledge were examined as potential contributors to the participants’ English reading comprehension. Given the results from the previous correlational analyses, the relationships between acculturation, vocabulary, and reading comprehension were homogeneous for the CG and EAP groups (see Table 6).

Two hierarchical multiple regression analyses were conducted using the results from W-J Passage comprehension as the dependent variable. The acculturation measure was entered as the first step, and the vocabulary test was entered as the second step. To further explore the unique contributions of the variables that are linked to reading comprehension, the order of the steps for each analysis was reversed (see Table 7). The results from the hierarchical regression analysis revealed that the independent variables explained 77.7% of the total variance for the passage comprehension, *R*^2^ = .777, *F*(2, 23) = 40.00, *p* < .001. The first model, with acculturation only, accounted for 51.1% of the variance, while vocabulary knowledge contributed 26.6% unique variance to reading comprehension. The sequence of steps in the model was reversed to ensure the completeness of the analysis. After reversing the order of variables entered in the hierarchical analysis, the vocabulary knowledge accounted for 58.8% of the variance as the first step in the model, while acculturation contributed 18.9% unique variance as the second step in the model.

**Table 7.** Predictors of passage comprehension using vocabulary and acculturation measures.

Variable			
Total *R*^2^ = .777	β	*t*	Δ*R*^2^
Step 1			.511
Acculturation	.715***	5.004	
Step 2			.266
Acculturation	.478***	4.413	
Vocabulary knowledge	.567***	5.235	
Step 1			.588
Vocabulary knowledge	.767***	5.848	
Step 2			.189
Vocabulary knowledge	.567***	.567	
Acculturation	.478***	4.413	

*** p < .001

### What are English learners’ experiences with different teaching methods and social interactions in Canada?

To explore the newcomers’ personal experiences learning English in each context, the quantitative and qualitative responses for both EAP and CG groups were examined. A common thread emerged among the participants regarding each question. A summary of these themes is reported below.

#### Experience with different instructional methods

The participants were asked about their perceptions of the value of the classes they had attended after arriving in Canada. Participants attended ESL classes in the community or EAP courses provided at a higher education institution. The participants were also asked about any improvements in their English skills after attending those classes. To analyze the participants’ opinions about their improvements after participating in either of the learning contexts, the participants’ responses were coded as a four-point Likert-type scale, with one representing “*not useful at all*” and four being “*very useful*.” A one-way ANOVA was conducted using the pedagogical method (i.e., CG or EAP) as the independent variable, while the perceived usefulness of the classes was the dependent variable. The analyses showed no significant differences in perceived usefulness between the EAP (*M* = 3.11, *SD* = .78) and CG learners (*M* = 2.80, *SD* = 1.23).

In addition to the quantitative analyses, the participant’s qualitative responses revealed nuances about the perception of usefulness and the notion of improvement based on their goals in learning English. For instance, many participants reported that CG-ESL courses helped them improve their speaking and listening skills. However, several participants in the CG reported that CG-ESL courses were not helpful for them in terms of English writing or reading comprehension. In addition, the participants’ goals for English learning emerged as an essential factor regarding their satisfaction with different teaching methods. For example, the participants in the CG, whose goal was improved social interaction without any specific aim to attain postsecondary education, were satisfied with CG-ESL courses. However, the individuals who reported that their goal was to pursue higher education reported less satisfaction with their experience in community ESL classes. Participants’ responses about the extent of perceived helpfulness were coded on a four-point scale, ranging from “*not helpful at all*” to “*very helpful*.” Fifty percent of the respondents with social integration goals for learning English found the community ESL classes to be “very helpful,” while another 37% found it to be slightly helpful. On the contrary, only 12% of the respondents with academic goals found the ESL classes “very helpful.” In comparison, 62% of those respondents in these courses reported that the classes were “slightly helpful.”

#### Perceived Effects of Knowledge of English Skills Based on Pedagogical Methods

To address this aspect of the study, the participants were asked to rate their perceived English language skills, consisting of speaking, listening, reading comprehension, and writing. Their perceived skills for each domain were rated on a scale from 0 (*no proficiency at all*) to 10 (*complete proficiency*). A one-way ANOVA was conducted with pedagogy type (i.e., CG-ESL or EAP courses) entered as a factor, and each language skill was added as a dependent variable. There was a significant difference between the students from the two different pedagogical methods, with EAP students rating their language proficiencies significantly higher for all the skills except for reading comprehension, *F*(1, 27) = .476, *p* = .496 (see Table 8).

**Table 8.** Between-subject comparisons for variables related to the participants’ perceived English skills.

	English for Academic Purposes Group		Community Group		
	*M*	*SD*	*M*	*SD*	*F*
Perceived usefulness of pedagogical methods	3.11	0.78	2.8	1.23	0.476
Perceived English-speaking skills	8.76	1.68	7	1.85	13.593**
Perceived English-listening skills	8.89	1.47	7.69	2.32	5.37**
Perceived English-writing skills	8.27	2.08	6.38	2.33	10.07**
Perceived English-reading comprehension skills	8.93	1.51	8.15	1.89	2.86

* *p* < .05. **p < .01.

#### Perceived effects of knowledge of English skills based on social integration

To provide additional context, the participants were asked to explain how their knowledge of the English language had affected their integration into society. In the CG group, 81% of the respondents reported that language skills in English were helpful for their community integration. For instance, some of the responses included: “It had a direct impact on the exchange of culture and the creation of new friendships” or “Sometimes, I feel I cannot express myself properly, or I feel shy when I have to little think (sic) before talking.” Only 43% of respondents in the EAP group stated that language has affected their integration into society. Conversely, 57% of the participants in the EAP group and only 19% of the respondents in the CG group reported that English was not a barrier to their integration. It is also important to note that the underlying reason for the lack of interference caused by English knowledge differed between the two groups. The participants in the CG group who did not find English skills necessary for day-to-day interactions reported that they mainly interacted within smaller communities consisting of individuals from their heritage culture. For example, a participant responded: “Where I live, most [people] speak fluent Farsi; I do not engage with my [English speaking] community whatsoever.” However, all the participants in the EAP group who did not find the language a barrier stated that their English is at a level where it does not cause any issues for them in communicating and integrating with society. For example, one participant stated: “No, my English is good enough that it has never affected my engagement with Canadians.”

## Discussion

The current study’s findings demonstrated similarities and differences between the two groups. For instance, acculturation was significantly related to English language skills for both groups. As a general theme, quantitative data revealed a significant advantage in language proficiency for EAP participants, followed by their higher acculturation scores. In addition, qualitative results suggest that participants in EAP courses reported a greater sense of integration and confidence in their language use, underscoring the value of academic settings in facilitating language learning and cultural adaptation.

The participants enrolled in EAP courses had significantly higher English skills than the community English learners. However, the findings also suggest that although there were differences among the learners’ academic, ethnic, and socio-cultural backgrounds, both learner groups benefit from pedagogical approaches tailored to their unique needs. Furthermore, these differences could partially relate to the participants’ motivation to learn English.

Another difference between the two groups was regarding acculturation and heritage enculturation levels. The participants enrolled in EAP courses had higher levels of acculturation than the community learners. These trends were in the opposite direction in terms of heritage enculturation. The community adult ESL learners had significantly higher heritage enculturation levels than EAP learners. The difference in acculturation between the two groups may be due to the community learners’ lower English skills, resulting in less interaction with Canadian culture. Acculturation was a significant predictor of English reading comprehension skills. One explanation for the university participants’ higher acculturation levels could be due to the environment in which they are primarily engaged. EAP learners were more likely to be exposed to other students at the university, including native English speakers. The possibility of more interaction with other individuals with better English language skills aligned with the interactional feedback process. Exposure to Canadian culture may have promoted a higher acculturation rate while on a university campus, with more exposure to the English-speaking communities, which likely contributed to the EAP group’s higher English language skills. In contrast, the CG learners were mainly in contact with peers who were also newcomer adult ESL learners.

Despite group differences in overall performance levels for most of the measures, the groups showed similarities in relationships among variables. When patterns of relationships were examined for the groups separately, they were similar, with differences likely due only to the sample size constraints. Consequently, subsequent analyses of the relations among variables were examined for the entire sample. Both vocabulary knowledge and acculturation were related to reading comprehension when the performance of the groups was examined together. Vocabulary was expected to be related to reading comprehension based on previously tested models (Gough & Tunmer, 1986). However, the contribution of acculturation to reading comprehension is less consistent in the literature and might result from the participants’ status as newcomers (Jia et al., 2014).

The findings from the qualitative analyses also demonstrated the mosaic nature of Canadian society. Although we have discussed the importance of acculturation for successful integration, some individuals may not necessarily find adjusting to mainstream culture important. As evident from the participants’ responses, several participants reported that despite a lack of fluency in English, they were able to meet their needs regarding daily life demands and socialization. These individuals may remain in their cultural group within Canadian society. While acculturation and language learning are essential for facilitating integration, some individuals prioritize their own cultural group over others. This highlights the importance of recognizing and respecting cultural diversity within Canada.

Another interesting finding was related to the participants’ acculturation, and how it was affected by their perception of fluency, independent of their L2 skills. The study revealed that participants who perceived lower English language skills as an obstacle to their acculturation process reported lower levels of integration and acculturation. This finding underscores the crucial role that language instructors can play in helping learners assess their language skills and boost their self-confidence to take risks in using their new language. By improving their English proficiency, individuals can become more involved in mainstream cultural activities and continue to refine their language skills. Promoting greater confidence in using their English language skills may enhance learners’ involvement with the host country’s mainstream culture, and consequently, their L2 skills may improve due to increased exposure to contextualized English language use in society. Although the qualitative results provided insights into relations among the participants’ language knowledge, preferences, and the perceived value of gains from English instruction, the number of respondents to these questions was limited. Therefore, further research may be required to better understand the interplay among different variables. The detailed qualitative analyses conducted for this study reveal the complex and diverse nature of Canadian society.

It is worth noting that acculturation and language acquisition processes are not straightforward and can be influenced by factors besides the variables examined in this study. Therefore, further research is necessary to fully comprehend the intricate interplay among these variables and their impact on acculturation in Canada. These analyses provide valuable insights into the complex nature of integration and highlight the need for ongoing efforts to understand and promote diversity and inclusivity in Canadian society.

The findings from the present study offer further information about English learners, their backgrounds, and the learning methods they found useful, including levels of English proficiency and satisfaction with course content. The qualitative questionnaire sheds further light on the English learners’ experiences and methods they found helpful. The qualitative analyses suggest that, independent from the learners’ objective improvement, both learning streams align with the specific participants’ goals. This finding provides additional evidence about the importance of appropriate guidance provided by educators or institutions based on the learners’ goals. Guiding the learners toward the learning methods compatible with their goals could avoid frustration and improve their satisfaction with the learning method and context to which they were exposed.

The current study also elicited the English learner’s actual vocabulary and reading comprehension proficiency based on appropriate standardized experimental measures to assess their language skills in English. The results favored the EAP learners regarding different aspects of language proficiency. However, the results should be interpreted with caution, as the EAP students may have had higher L1 and L2 skills prior to starting their classes in Canada.

This study provides further information about the learners’ motivation for learning English. Whether they have chosen the academic path or English for daily use class, the results provide further information on how to foster the appropriate conditions for learning based on the learners’ short-term and long-term language goals. Furthermore, the results suggest the potential role of applying what they have learned to settings outside of the classroom. The individuals who learned English in community colleges or universities showed higher English language skills. These results may be explained by the possibility that when students attend classes in these settings, they may interact more with native English speakers.

Conversely, the learners attending the community classes had fewer interactions with native English speakers within or outside of their environment. This is supported by the qualitative results, where the participants surrounded by individuals from their communities reported fewer interactions outside of their heritage language and culture. In contrast, EAP learners in the university setting were more likely to be exposed to individuals who were affiliated with the mainstream culture rather than to other newcomer immigrants who may lack exposure to the mainstream Canadian culture.

Furthermore, this study’s findings suggest that the context of ESL instruction plays a crucial role in not only language acquisition but also in the broader process of acculturation among adult newcomers. This finding underscores the importance of considering goals beyond language instruction and incorporating elements of cultural immersion and social integration when designing ESL programs.

## Implications

The implications of our study extend beyond academic discourse, offering policymakers actionable insights into optimizing ESL programs. For example, the differences observed regarding the relationship between acculturation rates and language proficiency suggest more diversified language instruction models for immigrant groups. Academic institutions and language instructors could benefit from more awareness about the socio-cultural dynamics at play by adapting methodologies to facilitate acculturation and language instruction. In addition, our findings suggest that the context of ESL instruction plays a crucial role not only in language acquisition but also in the broader process of acculturation among adult newcomers. For instance, although community-based instruction, such as LINC classes, does include culturally relevant materials, such as job postings or other elements to integrate real-life examples with English language skills, the instruction is limited within the institution, where, except for their interaction with the ESL instructor, the learners are only exposed to other non-proficient ESL learners. Based on the findings from this study, it would be ideal to encourage more real-life interaction between ESL learners and native English speakers in Canada to promote improved English language proficiency and acculturation among newcomer immigrants.

## Limitations and future directions

Although this study offers significant insights into the relationship between acculturation and second language acquisition among immigrants in Canada, there are some limitations that need to be considered. The snowball sampling methodology and the self-reported portion of the data may affect the generalizability of our findings. Future research could include further longitudinal designs to capture the evolution of language acquisition over time. Another limitation was the relatively small number of participants included in the study. Future studies with a larger sample size would be helpful to enhance the representativeness of the sample and examine the relationships further. Moreover, investigating the interplay between language learning and acculturation in other provinces with potentially different immigration landscapes and population demographics could provide a comparative perspective on the integration of adult ESL learners from a broader Canadian perspective.

## Conclusion

The current study has demonstrated the importance of considering different language instructional methods among newcomer immigrants to Canada. The study adds to the literature on adult ESL learners, especially those learning through different pedagogical methods. The finding underscores the importance of acculturation in acquiring English language skills despite differences in skill levels. Differences and similarities in language skills and learner goals within and across groups of learners should be considered when creating and selecting programs for adult ESL learners. The findings can assist policymakers and language institutions in better understanding the needs of L2 learners by emphasizing the importance of acculturation and informal interactions with native speakers in increasing L2 skills. Our research findings provide a more nuanced view of ESL education within the context of a globalized learning environment in Canada as an immigration country. It contributes to a deeper understanding of how adult ESL learners in Canada with diverse backgrounds can best be supported through tailored instructional methods that facilitate language learning and acculturation processes for better social adjustment and academic achievement within Canada.

## References

[bibr1-13670069251357191] Akamatsu N. (1999). The effects of first language orthographic features on word recognition processing in English as a second language. Reading and Writing, 11(4). 10.1023/A:1008053520326

[bibr2-13670069251357191] Baynham M. Simpson J. (2010). Adult language education and migration: Challenging agendas in policy and practice. Routledge.

[bibr3-13670069251357191] Berns R. G. Erickson P. M. (2001). Contextual teaching and learning: Preparing students for the new economy. Educational Resources Information Center (ERIC), 5.

[bibr4-13670069251357191] Berry J. W. (1980). Acculturation as varieties of adaptation. In Acculturation: Theory, models and findings (pp. 9–25). Westview.

[bibr5-13670069251357191] Brown J. S. Collins A. Duguid P. (1989). Situated cognition and the culture of learning. Educational Researcher, 18(1), 32–42.

[bibr6-13670069251357191] Brunsting N. C. Smith A. C. Zachry C. (2018). An academic and cultural transition course for international students: Efficacy and socio-emotional outcomes. Journal of International Students, 8(4). 10.5281/zenodo.1467805

[bibr7-13670069251357191] California Department of Education. (2025). Facts about English learners in California. California Department of Education. https://www.cde.ca.gov/ds/ad/cefelfacts.asp

[bibr8-13670069251357191] Cuellar I. Arnold B. Maldonado R. (1995). Acculturation rating scale for Mexican Americans-II: A revision of the original ARSMA scale. Hispanic Journal of Behavioral Sciences, 17(3). 10.1177/07399863950173001

[bibr9-13670069251357191] Dietrich S. Hernandez E. , & U.S. Census Bureau. (2022). Language use in the United States: 2019 (ACS-50). https://www.census.gov/content/dam/Census/library/publications/2022/acs/acs-50.pdf

[bibr10-13670069251357191] Durgunoǧlu A. Y. (2002). Cross-linguistic transfer in literacy development and implications for language learners. Annals of Dyslexia, 52. 10.1007/s11881-002-0012-y

[bibr11-13670069251357191] Gough P. B. Tunmer W. E. (1986). Decoding, reading, and reading disability. Remedial and Special Education, 7(1). 10.1177/074193258600700104

[bibr12-13670069251357191] Government of Canada. (2018). ARCHIVED–Backgrounder–Language Instruction for Newcomers to Canada (LINC) Program. https://www.canada.ca/en/immigration-refugees-citizenship/news/archives/backgrounders-2013/language-instruction-newcomers-canada-linc-program.html

[bibr13-13670069251357191] Holec H. (1981). Autonomy and foreign language learning. In Kamberi F. (Ed.), Autonomia e Apprendimento Autodiretto Nella Prospettiva Europea. Consiglio d’Europa.

[bibr14-13670069251357191] Hymes D. (1966). Two types of linguistic relativity. In Bright W. (Ed.), Sociolinguistics (pp. 114–158). Mouton.

[bibr15-13670069251357191] Isphording I. E. (2014). The intergenerational transmission of language skills and the role of parental education. Review of Economics of the Household, 12(3), 407–429.

[bibr16-13670069251357191] Jackson J. Howard M. Schwieter J. W. (2020). Language proficiency Developmental perspectives and linguistic outcomes of education abroad. In Education abroad: Bridging scholarship and practice. Routledge.

[bibr17-13670069251357191] Jackson J. Schwieter J. W. (2019). Study abroad and immersion. In Schwieter J. W Benati A. (Eds.), The Cambridge handbook of language learning (pp. 727−750). Cambridge University Press.

[bibr18-13670069251357191] Jia F. Gottardo A. Koh P. W. Chen X. Pasquarella A. (2014). The role of acculturation in reading a second language: Its relation to English literacy skills in immigrant Chinese adolescents. Reading Research Quarterly, 49(2). 10.1002/rrq.69

[bibr19-13670069251357191] Jiang M. Green R. J. Henley T. B. Masten W. G. (2009). Acculturation in relation to the acquisition of a second language. Journal of Multilingual and Multicultural Development, 30(6). 10.1080/01434630903147898

[bibr20-13670069251357191] Kosyakova Y. Kristen C. Spörlein C. (2022). The dynamics of recent refugees’ language acquisition: How do their pathways compare to those of other new immigrants? Journal of Ethnic and Migration Studies, 48(5). 10.1080/1369183X.2021.1988845

[bibr21-13670069251357191] Mickan A. McQueen J. M. Lemhöfer K. (2019). Bridging the gap between second language acquisition research and memory science: The case of foreign language attrition. Frontiers in Human Neuroscience, 13. 10.3389/fnhum.2019.00397 PMC6866255 31798433

[bibr22-13670069251357191] Mitchell R. Myles F. Marsden E. (2013). Second language learning theories. In Second Language Learning Theories. Taylor & Francis. 10.4324/9780203770795

[bibr23-13670069251357191] Mustafa M. C. Radzi N. M. M. Masnan A. H. Bacotang J. Isa Z. M. Osman Z. Alias A. (2019). Teacher practices in the acquisition of English among Asian immigrant English language learners. Malaysian Journal of Learning and Instruction, 16(1). 10.32890/mjli2019.16.1.9

[bibr24-13670069251357191] Nassaji H. (2015). Interactional feedback dimension in instructed second language learning. Bloomsbury Academic.

[bibr25-13670069251357191] Nation P. Beglar D. (2007). A vocabulary size test. The Language Teacher, 31(7), 9–13. http://www.jalt-publications.org/archive/tlt/2007/07_2007TLT.pdf

[bibr26-13670069251357191] Nieuwboer C. van’t Rood R. (2016). Learning language that matters. A pedagogical method to support migrant mothers without formal education experience in their social integration in Western countries. International Journal of Intercultural Relations, 51. 10.1016/j.ijintrel.2016.01.002

[bibr27-13670069251357191] Purcell-Gates V. Degener S. C. Jacobson E. Soler M. (2002). Impact of authentic adult literacy instruction on adult literacy practices. Reading Research Quarterly, 37(1). 10.1598/rrq.37.1.3

[bibr28-13670069251357191] Riazi A. Farsani M. (2024). Mixed-methods research in applied linguistics: Charting the progress through the second decade of the twenty-first century. Language Teaching, 57(2), 143–182.

[bibr29-13670069251357191] Riches C. Genesee F. (2006). Literacy: Crosslinguistic and crossmodal issues. In Educating English language learners: A synthesis of research evidence. 10.1017/CBO9780511499913.004

[bibr30-13670069251357191] Schumann J. H. (1986). Research on the acculturation model for second language acquisition. Journal of Multilingual and Multicultural Development, 7(5). 10.1080/01434632.1986.9994254

[bibr31-13670069251357191] Schwieter J. W. Ferreira A. (2020). Learning a language abroad and the implications for social participation and positioning. Critical Inquiry in Language Studies, 17(1). 10.1080/15427587.2020.1713785

[bibr32-13670069251357191] Schwieter J. W. Howard M. Jackson J. (Forthcoming). Learning context: Study abroad and immersion in second language acquisition. In Leeser M. Sunderman G. (Eds.), Second language acquisition. Bloomsbury.

[bibr33-13670069251357191] Schwieter J. W. Jackson J. Ferreira A. (2021). When “domestic” and “international” students study abroad: Reflections on language learning, contact, and culture. International Journal of Bilingual Education and Bilingualism, 24(1). 10.1080/13670050.2018.1447545

[bibr34-13670069251357191] Schwieter J. W. Kunert S. (2012). Short-term study abroad and cultural sessions: Issues of L2 development, identity, and socialization. In Chamness Miller P. Watze J. Mantero M. (Eds.), Readings in language studies: Vol. 3. Language and identity (pp. 587–604). International Society for Language Studies.

[bibr35-13670069251357191] Soto Huerta M. E. Pérez B . (2015). Second-language literacy, immigration, and globalization. International Journal of Bilingual Education and Bilingualism, 18(4). 10.1080/13670050.2014.928257

[bibr36-13670069251357191] Statistics Canada. (2017). Immigrant languages in Canada. https://www150.statcan.gc.ca/n1/pub/11-627-m/11-627-m2017025-eng.htm

[bibr37-13670069251357191] Statistics Canada. (2022). While English and French are still the main languages spoken in Canada, the country’s linguistic diversity continues to grow. The Daily. https://www150.statcan.gc.ca/n1/daily-quotidien/220817/dq220817a-eng.htm

[bibr38-13670069251357191] Steen S. Liu X. Shi Q. Rose J. Merino G. (2017). Promoting school adjustment for English-language learners through group work. Professional School Counseling, 21(1). 10.1177/2156759x18777096

[bibr39-13670069251357191] Vygotsky L. S. (1978). Mind in society: The development of higher psychological processes. Harvard University Press.

[bibr40-13670069251357191] Woodcock R. McGrew K. Mather N. (2001). Woodcock-Johnson III tests of achievement. Riverside Publishing.

[bibr41-13670069251357191] Young T. J. Schartner A. (2014). The effects of cross-cultural communication education on international students’ adjustment and adaptation. Journal of Multilingual and Multicultural Development, 35(6). 10.1080/01434632.2014.884099

[bibr42-13670069251357191] Zhang K. Sun X. Flores-Gaona Z. Yu C.-L. Rachel L. Eggleston R. Nickerson N. Caruso V. Tardif T. Kovelman I. (2024). Cross-linguistic transfer in bilingual children’s phonological and morphological awareness skills: A longitudinal perspective. Bilingualism: Language and Cognition, 28, 327–342. 40331214 10.1017/s1366728924000439 PMC12052307

